# Computational identification of necroptosis-related hub genes and therapeutic targets in dilated cardiomyopathy via integrated analysis of bulk and single-cell RNA sequencing public cohorts

**DOI:** 10.1097/MD.0000000000046959

**Published:** 2026-01-02

**Authors:** Meng Xia, Yanan Ji, Hongtao Zhu

**Affiliations:** a Faculty of Biochemical and Environmental Engineering, Baoding University, Baoding, China; b Department of Cardiology, Danyang Hospital Affiliated to Nantong University, Danyang, China.

**Keywords:** dilated cardiomyopathy, machine learning, necroptosis, single-cell sequencing, therapeutic drug

## Abstract

Dilated cardiomyopathy (DCM) is a prevalent myocardial disease with a grim poor prognosis, and its pathogenesis remains poorly understood. Necroptosis, a programmed cell death mechanism, is believed to be significantly involved in the pathogenesis of DCM. This study aimed to identify necroptosis-related hub genes associated with DCM and predict potential agents for DCM. All analyses exclusively utilized publicly available datasets from the Gene Expression Omnibus database. Differentially expressed genes were identified in the DCM dataset GSE128095. The least absolute shrinkage and selection operator and random forest algorithms were then employed to identify 5 necroptosis-related hub genes. A diagnostic model for DCM was constructed based on these hub genes, and the expression profiles of these genes across different myocardial cell populations were systematically analyzed using single-cell RNA sequencing data from GSE184899 dataset. Furthermore, the drug-gene interaction database was utilized to identify potential therapeutic candidates targeting these hub genes. A total of 17 necroptosis-related genes were identified, primarily associated with biological processes such as apoptosis, antiviral immune response, and autophagy, with a particular localization in lysosome/autophagy-related structures and membrane functional regions. Kyoto Encyclopedia of Genes and Genomes pathway enrichment analysis revealed that these genes predominantly regulated the necroptosis and NOD-like receptor signaling pathways. Five necroptosis-related hub differentially expressed genes (*CAPN1*, *SLC25A5*, *IFNGR2*, *CAMK2A*, and *BIRC3*) were pinpointed. The DCM diagnostic model based on these 5 hub genes demonstrated excellent diagnostic efficacy. These hub genes displayed distinct expression profiles across various cardiac cell types. Notably, the upregulated hub genes in DCM including *SLC25A5*, *IFNGR2*, and *CANP1*, exhibited increased expression in multiple cardiac cell types, encompassing cardiomyocytes, smooth muscle cells, and endothelial cells. Conversely, the downregulated gene *CAMK2A* in DCM displayed a specific expression preference in fibroblasts. Finally, Calpeptin was predicted as a potential therapeutic agent for DCM. This study computationally identified 5 hub genes associated with necroptosis in DCM, developed a diagnostic model, and proposed one potential therapeutic drug, providing novel insights for the diagnosis and management of DCM.

## 1. Introduction

Dilated cardiomyopathy (DCM) is a multifaceted myocardial condition characterized by ventricular enlargement, impaired contractility, and involvement of diverse etiologies and pathogenic mechanisms.^[1,2]^ In notable proportion of DCM patients, approximately 5% to 10%, experience atrial fibrillation, significantly increasing the risks of thromboembolic events and sudden cardiac death.^[3]^ Despite improvements in patient prognosis in recent years, DCM remains a primary contributor to heart failure and premature mortality.^[2]^ Direct diagnosis of DCM using a single test is challenging. To enhance diagnostic specificity and accuracy, a combination of imaging modalities and biomarkers is necessary, involving the systematic exclusion of secondary factors and integration of multi-dimensional data. Current single-cell sequencing studies have identified notable transcriptomic differences between DCM and hypertrophic cardiomyopathy.^[4]^ However, significant gaps persist in understanding the specific molecular mechanisms of DCM, particularly with regard to the precise mechanism of cardiomyocyte death and its correlation with disease progression.^[5]^

Recent research has confirmed the significance of necroptosis, a form of programmed necrosis regulated by the RIPK1/RIPK3/MLKL signaling pathway,^[6,7]^ in various cardiovascular conditions such as myocardial infarction, ischemia-reperfusion injury, heart failure, and DCM.^[8–13]^ Distinct from apoptosis, necroptosis causes morphological changes in cardiomyocytes resembling necrosis and initiates inflammation in adjacent tissues, leading to myocardial injury, fibrosis, and adverse remodeling.^[14]^

Research in DCM has highlighted the pivotal role of genetic factors. Specifically, the deletion or mutation of the *TAB2* gene has been associated with DCM pathogenesis by activating the RIPK1-dependent pathway, leading to apoptosis and necroptosis.^[11]^ Similarly, *TEAD1* deficiency triggers the necroptotic apoptosis pathway, resulting in extensive cardiomyocyte death and a pronounced inflammatory response.^[9]^ Furthermore, mutations in synaptic *Nesprin-1/-2* genes have been linked to necroptosis induction through the disruption of nuclear membrane mechanotransduction.^[9]^ Collectively, these studies underscore the significance of necroptosis as a fundamental mechanism underlying pathological damage in DCM. The precise involvement of necroptosis in DCM remains inadequately characterized, highlighting the urgent need to elucidate its molecular and cellular mechanisms to identify potential therapeutic targets for disease management and improvement of prognosis. Investigating the clinical diagnostic potential of necroptosis through transcriptome sequencing to analyze the distinct transcriptomic profiles of necroptosis-associated cells within myocardial tissues is essential to enhance understanding of DCM pathogenesis. Rapid advances in bioinformatics have accelerated the process.^[15]^

In this study, the bulk-seq data of DCM and normal endocardial myocardial biopsy samples from the GEO database were processed using R software to identify differentially expressed genes (DEGs). An intersection analysis was performed between necroptosis-related genes from the Kyoto Encyclopedia of Genes and Genomes (KEGG) database, followed by functional enrichment analysis of the overlapping genes. The study employed Least Absolute shrinkage and Selection Operator (LASSO) and random forest (RF) machine learning algorithms to narrow down hub genes associated with necroptosis in DCM. A diagnostic model based on 5 hub genes was developed and assessed for diagnostic efficacy. Additionally, an initial assessment of cell localization and expression patterns of these hub genes was conducted using single-cell RNA sequencing (scRNA-seq) data, revealing a specific cell subpopulation potentially contributing to DCM pathogenesis. The Drug-Gene interaction database (DGIdb) was employed to predict potential therapeutic drugs for DCM, establishing a link between relevant genes and drugs to provide insights for clinical precision treatment and experimental investigations in DCM.

## 2. Materials and methods

### 2.1. Data acquisition and differential gene analysis

The GSE128095 dataset was used for transcriptome analyses from endomyocardial biopsies of 47 DCM patients presenting with LVEF (left ventricular systolic dysfunction) < 45% and New York Heart Association functional class II and III heart failure symptoms, along with 8 individuals with normal LVEF. Differential gene expression analysis between the DCM patient group and the control group was conducted using the “limma” package [3.60.4]^[16]^ with a significance threshold set at *P* value < .05. The results of the differentiation analyses were displayed as heat maps and volcanic graphs.

### 2.2. Screening for necroptosis-related DEGs

Necroptosis-related genes were obtained from the KEGG database. The necroptosis-related DEGs were determined by intersecting the DEGs with necroptosis genes using the “VennDiagram” package [1.7.3] in R.

### 2.3. Functional enrichment analysis

The GO (Gene Ontology) and KEGG pathway enrichment analyses were performed on the selected necroptosis-related DEGs through the “clusterProfiler” software package [4.12.4] in R.^[17]^ The threshold of statistical significance was established at *P* < .05.

### 2.4. Identification of necroptosis-related hub DEGs

The LASSO and RF methods were employed to identify the critical genes. LASSO analysis was performed using the “glmnet” package [4.1.10] in R.^[18]^ Through conducting 10-fold cross-validation on a range of candidate lambda values, the prediction errors of various models corresponding to different lambdas are assessed. Subsequently, the optimal parameter, lambda. min, is identified as the lambda value that minimizes the mean cross-validation error. The RF model was constructed using the “randomForest” package [4.7.1.1] in R.^[19]^ The analysis utilized 500 decision trees with default mtry settings and identified key variables based on average Gini importance for further investigation. Core genes for mechanistic exploration and predictive modeling were selected by integrating model accuracy, variable importance scores, and cross-validation outcomes. The necroptosis-related hub DEGs were identified by intersecting the results from the LASSO and RF algorithms using a Venn diagram. To evaluate the diagnostic efficacy of each gene, the receiver operating characteristic (ROC) curves were generated, and the area under the curve (AUC) values were calculated in the GSE120895 dataset using the “pROC” package [1.18.0] in R.^[20]^ An AUC exceeding 0.7 indicated satisfactory discriminatory performance, while a *P* value less than .05 denoted statistical significance.

### 2.5. Establishment of diagnostic model for DCM

The diagnostic model was established to evaluate the diagnostic efficacy of necroptosis-related hub DEGs using the “rms” package [6.4.0] in R. The ROC, calibration curves, and the subsequent decision curve analysis (DCA) clinical benefit curve were then used to assess the reliability of the model predictions. The “pROC” package [1.18.0] was used for ROC curve plotting, and the “rmda” package [1.6] was used to draw the DCA clinical benefit curve.

### 2.6. Clustering and annotation of single-cell RNA sequencing data

Single cell cluster analysis was performed on the GSE184899 dataset using “Seurat” package [4.3.0] in R.^[21–25]^ The GSE184899 dataset comprises scRNA-seq data from human induced pluripotent stem cell-derived cardiomyocytes (iPSC-CMs) sampled from a congenital dilated cardiomyopathy iPSC line and a control iPSC line at differentiation day 35. Analysis followed standard Seurat procedures. Initially, quality control measures were applied to the original expression matrix, retaining only high-quality cells with a gene count ranging from 200 to 6000 and a mitochondrial gene proportion below 10% for subsequent analysis. Normalization of the data was performed using the LogNormalize method with a normalization factor of 10,000. The top 2000 hypervariable genes were identified using the FindVariableFeatures function for principal component analysis. Subsequently, Z-score normalization of all genes was carried out using the ScaleData function, followed by principal component analysis using RunPCA with the selection of the first 30 principal components for clustering and dimension reduction. Cell clustering was conducted by constructing an SNN map with the FindNeighbors and FindClusters functions, setting the cluster resolution at 0.5. Visualization of cell data in 2 dimensions was achieved using the RunTSNE function for subgroup identification and labeling. Known immune cell markers such as *CD3D*, *CD14*, *MS4A1*, *NKG7*, and *PPBP* were utilized in conjunction with t-SNE (t-distributed Stochastic Neighbor Embedding) distribution and differential expression results for cell type annotation. Differential expression analysis was performed through the Wilcoxon rank sum test with a screening threshold defined as an absolute log2 fold change greater than 0.25, a *P* value below .05 after correction, and a minimum percentage greater than .1. The identified marker genes were extracted to represent different cell groups. Simultaneously, the IntegrateData function is employed when dealing with multiple batches or samples to merge diverse data sources and mitigate potential batch effects. Moreover, cell cycle scoring and the DoubleFinder algorithm are applied to enhance data quality by identifying and eliminating double cells.

### 2.7. Predicting potential drugs

The DGIdb was used to predict the potential drugs targeting 5 necroptosis-related hub DEGs.

### 2.8. Statistical analysis

**Figure 1.**
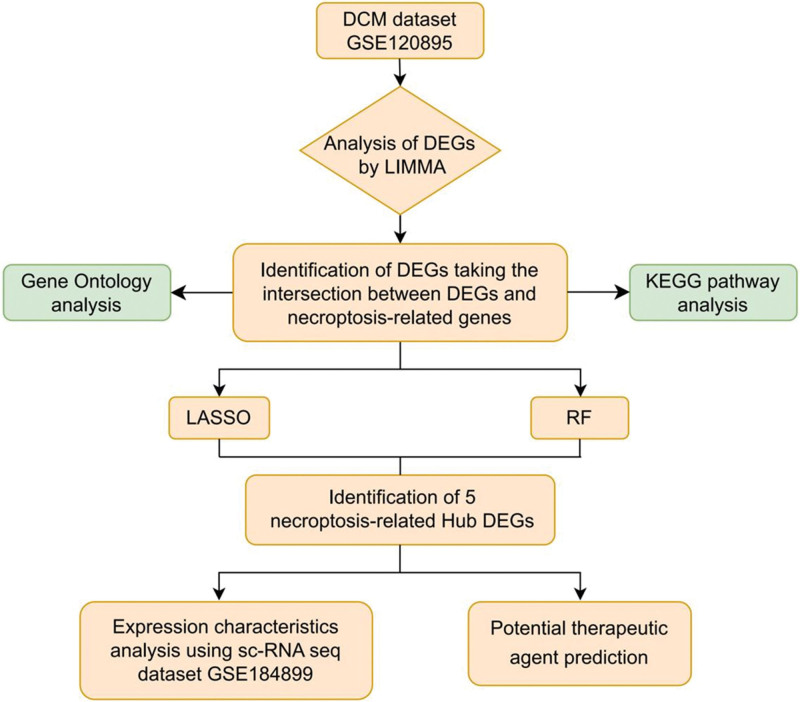
Flowchart of the study design.

All statistical analyses were conducted using the R software package. Group comparisons were assessed using the t-test or Wilcoxon rank sum test. All statistical tests were two-tailed, and *P* value < .05 was considered statistically significant. The study’s flowchart is depicted in Figure 1.

## 3. Results

### 3.1. Identification of DEGs

**Figure 2.**
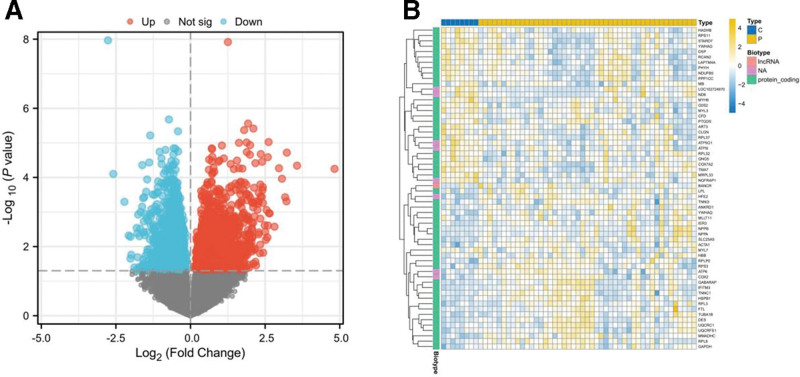
Identification of DEGs in GSE120895. (A) The volcano plot of DEGs (*P* value < .05). (B) The heatmap of DEGs. DEGs = differentially expressed genes.

GSE120895 from the GEO dataset contained 12,412 genes analyzed across 55 samples of endocardium myocardium, comprising 47 DCM patients who had heart failure with reduced ejection fraction and 8 individuals with normal LVEF. Among the genes examined in GSE120895, a total of 3190 were identified as DEGs, with 1291 genes demonstrating upregulation and 1899 genes showing downregulation, as illustrated in a volcano plot (Fig. 2A). The top 30 DEGs were further visualized using a heatmap (Fig. 2B).

### 3.2. Screening and functional enrichment of necroptosis-related differentially-expressed genes (NRDEGs)

**Figure 3.**
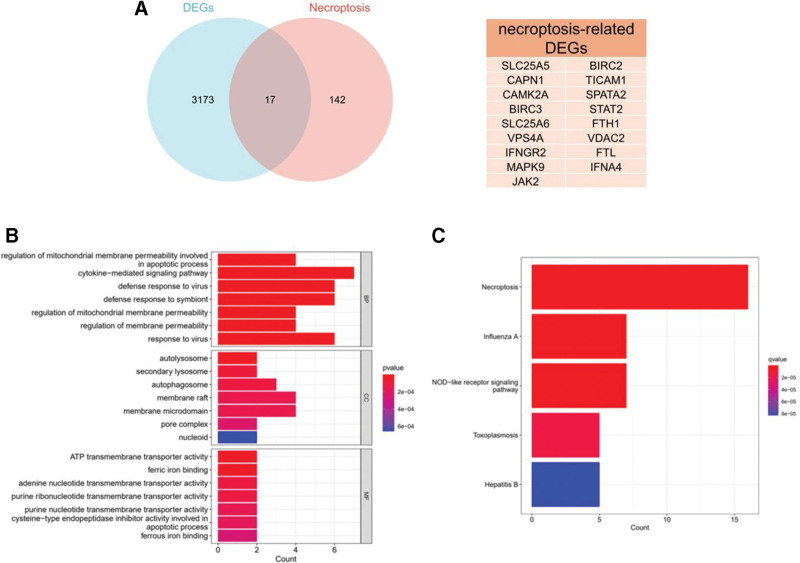
Screening and functional enrichment analysis of NRDEGs. (A) The Venn diagram demonstrating the interaction between DEGs and necroptosis-related genes. (B) GO enrichment analysis of NRDEGs, showcasing the top 7 significantly enriched terms (*P* value < .05) categorized into BP, MF, and CC. The intensity of bar color corresponds to the *P* value, while the length of the bar represents the number of genes associated with each term. (C) The KEGG analysis of NRDEGs, highlighting the top 5 significantly enriched signaling pathways (*P* value < .05). BP = biological process, CC = cellular component, GO = gene ontology, KEGG = Kyoto Encyclopedia of Genes and Genomes, MF = molecular function, NRDEGs = necroptosis-related differentially-expressed genes.

The 3032 DEGs overlapped with 159 necroptosis-related genes obtained from KEGG database, resulting in 17 shared genes, referred to as NRDEGs (Fig. 3A). These NRDEGs underwent GO functional enrichment and KEGG pathway analyses, revealing enrichment in 352 biological processes, 57 molecular functions, 20 cellular components, and 55 pathways (*P* value < .05). The GO analysis showed that NRDEGs were predominantly involved in apoptosis regulation, antiviral immune response, and autophagy. At the molecular level, they exhibited functions related to transmembrane transport activity and metal binding, while their cellular components were concentrated in lysosome/autophagy-related structures and membrane functional regions (Fig. 3B). KEGG pathway enrichment analysis showed that NRDEGs mainly regulated necroptosis and NOD-like receptor signaling pathway (Fig. 3C).

### 3.3. Identification of necroptosis-related hub DEGs and diagnostic prediction

**Figure 4.**
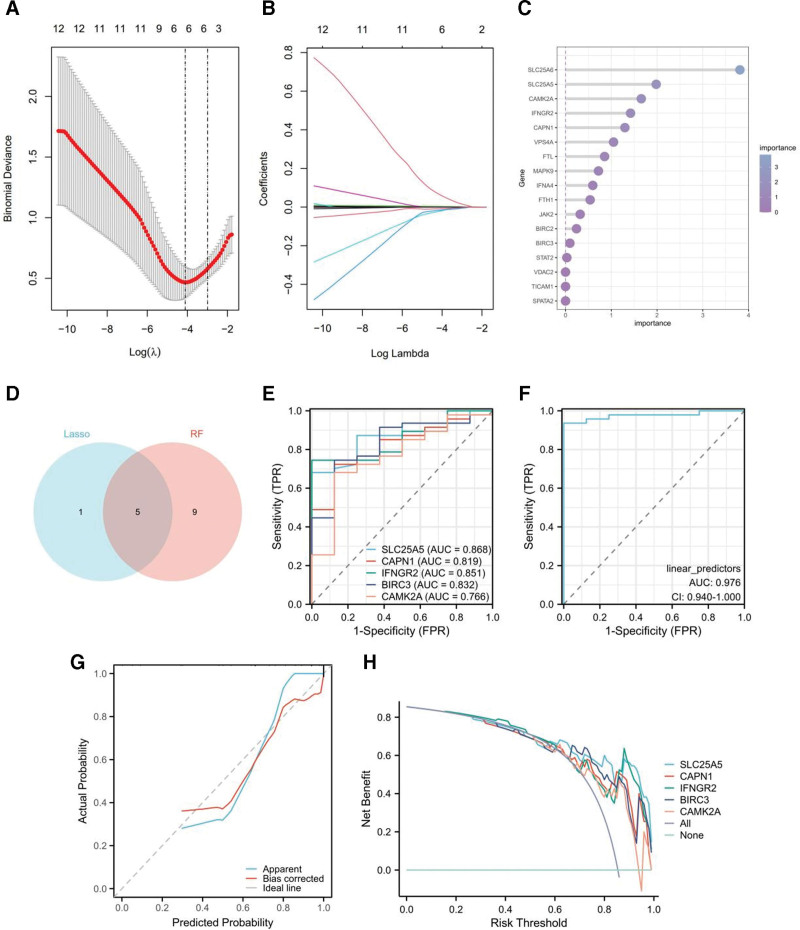
Identification and diagnostic evaluation of necroptosis-related hub DEGs. (A) LASSO logic coefficient penalty diagram. (B) The LASSO plot illustrates the variations in the size of coefficients for parameters shrank as the value of k penalty increased. (C) Identification of necroptosis-related hub DEGs based on variable importance assessed through RF analysis. (D) Venn diagram depicting the 5 common necroptosis-related hub genes identified by both LASSO and RF. (E) ROC curves for the 5 hub genes to evaluate their diagnostic utility. (F) ROC curve of the DCM risk model. (G) Calibration curves of the DCM risk models. (H) DCA for the DCM risk model. DCA = decision curve analysis, DCM = dilated cardiomyopathy, DEGs = differentially expressed genes, LASSO = least absolute shrinkage and selection operator, RF = Random Forest, ROC = receiver operating characteristic.

To further identify the more critical hub genes associated with DCM, we applied 2 different machine learning algorithms to obtain the most robust results. Through the LASSO algorithm, we identified 6 characteristic genes (Fig. 4A and B). Additionally, RF methodology was applied to prioritize variables, leading to the identification of the top 14 genes with importance scores above zero (Fig. 4C). Subsequently, 5 genes (*CAPN1*, *SLC25A5*, *INFGR2*, *CAMK2A*, and *BIRC3*) overlapped between LASSO and RF analysis were identified as necroptosis-related hub DEGs (Fig. 4D). The diagnostic efficacy of these 5 hub DEGs was evaluated using ROC curves, with each hub gene exhibiting an AUC value exceeding 0.7 (Fig. 4E). Therefore, these hub genes demonstrate effective diagnostic capabilities for DCM. After the construction of the diagnostic model of the 5 hub DEGs, the dataset exhibited an AUC value of 0.976 (Fig. 4F). The calibration curve trend fitted well with the standard curve (Fig. 4G), indicating the reliability of the model. Furthermore, the DCA showed that the constructed diagnostic model can enhance the predictive ability for the risk of DCM occurrence within a certain risk threshold probability range (Fig. 4H).

### 3.4. Dataset validation

**Figure 5.**
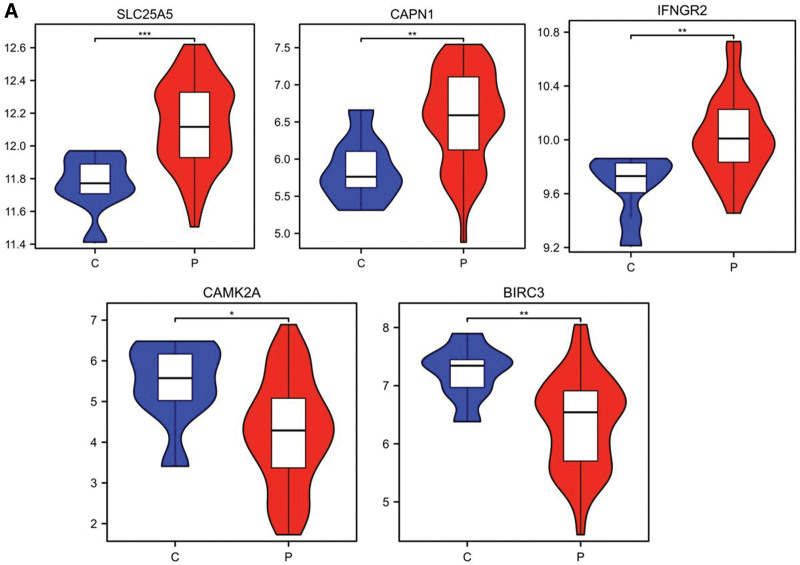
Expression levels of 5 necroptosis-related hub genes in GSE120895. (A) The violin plots depicting the gene expression levels of *SLC25A5*, *CAPN1*, INFGR2, CAMK2A, and BIRC3 in control (C) and patient (P) groups. *P < .05; **P < .01; ***P < .001.

In the GSE120895 dataset, the violin diagram illustrates that in comparison to the control group, the gene expression levels of *SLC25A5*, *CAPN1*, *IFNGR2* were upregulated in the DCM patient group, while the expression of *BIRC3* and *CAMK2A* was downregulated (Fig. 5A).

### 3.5. Expression characteristics of necroptosis-related hub genes in different cardiac cell populations

**Figure 6.**
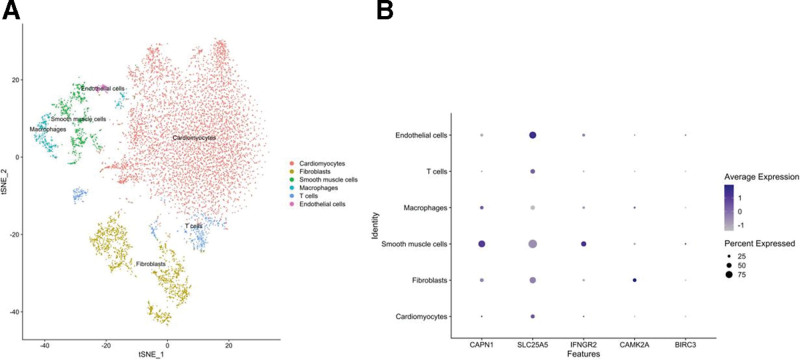
Single-cell clustering, cell type annotation and expression characteristics analysis of necroptosis-related hub genes. (A) t-SNE diagram depicting 6 cardiac cell clusters. (B) Dot plots showing the average expression and percentage of expression of 5 necroptosis-related hub genes across the 6 cardiac cell clusters. t-SNE = t-distributed Stochastic Neighbor Embedding.

To delve into the expression profiles of necroptosis-related hub genes across various cardiac cell populations and address cellular heterogeneity in Bulk-seq data, scRNA-seq data from the GSE184899 dataset was utilized for analysis. The tSNE diagram showed 6 spatially distinctly separated types of cardiac cells, including cardiomyocytes, macrophages, fibroblasts, smooth muscle cells, T cells, and endothelial cells, based on marker genes (Fig. 6A). Subsequently, average expression and percent expressed analysis of 5 necroptosis-related hub DEGs across these different cardiac cell populations were performed (Fig. 6B). In smooth muscle cells and cardiomyocytes, *CANP1* and *IFNGR2* exhibited comparatively elevated expression levels. *SLC25A5* showed enhanced transcriptional activity predominantly in endothelial cells and cardiomyocytes. These findings suggested that the upregulated hub genes associated with necroptosis exhibit functional significance not only in cardiomyocytes but also demonstrate conserved regulatory potential within smooth muscle cell and endothelial cells. Furthermore, *CAMK2A* exhibited a specific expression predominance in fibroblasts.

### 3.6. Potential therapeutic drug prediction

**Figure 7.**
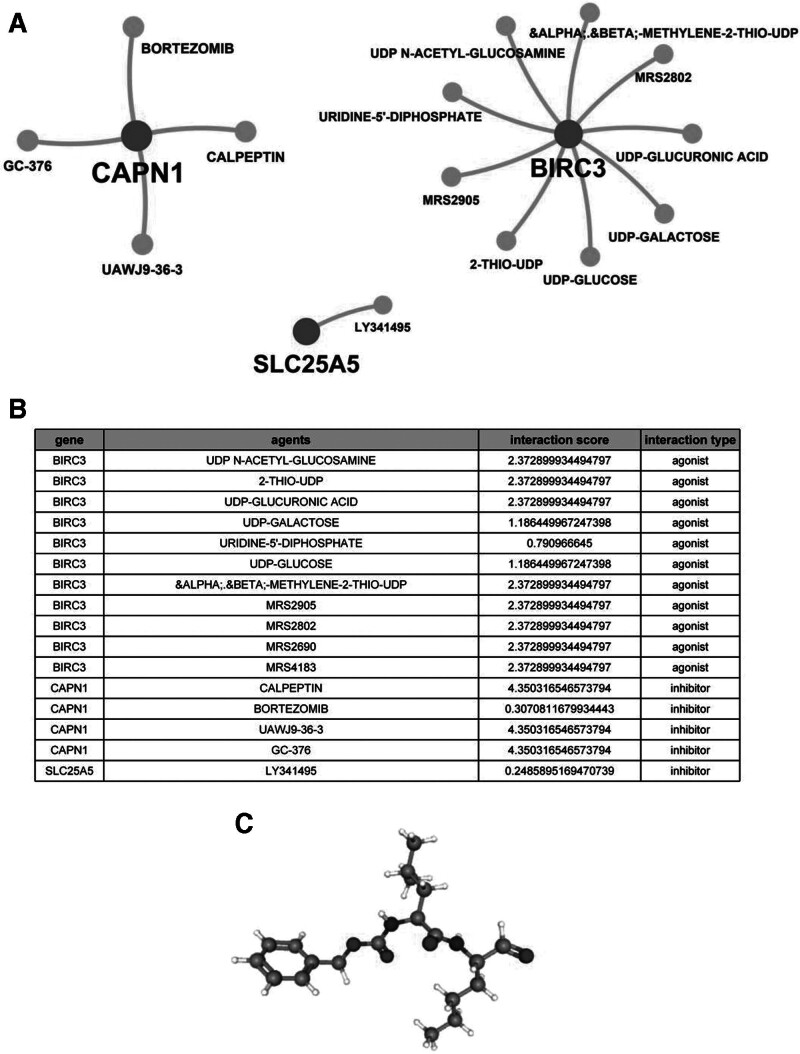
Screening potential therapeutic drugs for DCM. (A) Diagram of the drug-gene interaction network. (B) List of drug-gene interaction scores and interaction types. (C) Chemical structures of Calpeptin. DCM = dilated cardiomyopathy.

Utilizing a systematic drug discovery approach with DGIdb, we conducted a network pharmacology analysis to identify potential therapeutic drugs capable of modulating necroptosis in DCM. Our analysis generated an interactive drug-gene network highlighting promising candidates, including 4 CAPN1 inhibitors, eleven BIRC3 agonists, and one SLC25A5 inhibitor (Fig. 7A and B). Following evaluation of molecular mechanisms and functional annotations, a compound with notably high therapeutic potential emerged: Calpeptin. Calpeptin, a cell-permeable calpain inhibitor, exerts potent enzymatic inhibition by covalently modifying the calpain active site, thereby influencing critical pathways in cellular stress response^[26,27]^ (Catalano, Deaton et al 2004, Tabata, Tabata et al 2010). Studies have indicated that Calpeptin plays a protective role in cardiovascular pathological processes by inhibiting calpain activities^[28]^ (Mani, Shiraishi et al 2008). The chemical structure of Calpeptin was retrieved from the PubChem database for reference (Fig. 7C).

## 4. Disscussion

DCM is a prevalent condition associated with heart failure, arrhythmias, and sudden death, characterized by ventricular enlargement and reduced systolic function, contributing to a high mortality rate and a 5-year mortality rate ranging from 15% to 50%.^[1,29,30]^ Early diagnosis of DCM is crucial for enhancing patient prognosis. However, clinical manifestations of DCM often lack specificity, and patients may remain asymptomatic initially due to compensatory cardiac mechanisms, leading to potential underdiagnosis or misdiagnosis. The diverse etiology of DCM complicates diagnosis, necessitating a comprehensive approach involving imaging modalities, laboratory tests, genetic assessments, and endomyocardial biopsies for accurate evaluation.

Advancements in bioinformatics have significantly enhanced our comprehension of the molecular mechanisms involved in complex diseases, focusing researchers on the analysis of RNA-seq data to discover novel markers and therapeutic targets for DCM. While necroptosis has been linked to DCM development, research in this area remains limited, necessitating further exploration.^[8–11]^ To address this gap, we have selected a suitable gene chip to identify potential diagnostic biomarkers linked to necroptosis in DCM and elucidate their roles in the disease. Our aim is to provide clinically relevant findings to advance the treatment and prevention strategies for DCM.

In this investigation, we utilized R software to identify DEGs from bulk-seq data of DCM. Through the integration of this data with necroptosis databases, we initially identified 17 necroptosis-related genes. Subsequent GO and KEGG functional enrichment analyses revealed that these 17 genes primarily participate in processes related to mitochondria and cellular autophagy. We then employed 2 machine learning algorithms, LASSO and Random Forest, to refine the selection of necroptosis genes associated with DCM, resulting in the identification of 5 hub genes: *SLC25A5*, *CNAP1*, *IFNGR2*, *CAMK2A*, and *BIRC3*. By constructing a diagnostic model based on these 5 hub genes, our findings demonstrated predictive accuracy for diagnosing DCM. Furthermore, a preliminary cell localization and expression analysis was performed on these hub genes using sc-RNA seq data, revealing a subset of cells, including cardiomyocytes, endothelial cells, and smooth muscle cells, with potential contributions to DCM pathogenesis.

SLC25A5, also known as ANT2, is a member of the mitochondrial carrier subfamily of solute carrier protein genes that facilitate the exchange of ADP and ATP between the extramitochondrial and intramitochondrial compartments. Within this subfamily, there are 4 isoforms: ANT1, ANT2, ANT3, and ANT4. ANT1 is predominantly expressed in the heart and bone, ANT2 is primarily found in highly proliferative cells like cancer cells, hepatocytes, and neural stem cells, while ANT3 exhibits ubiquitous expression across tissues. ANT4 shows elevated expression levels in liver and testicular tissues. The various ANT isoforms play distinct roles in apoptosis, with ANT3 and ANT1 typically displaying pro-apoptotic properties, whereas ANT2 and ANT4 inhibit apoptosis, exerting a protective effect on cells.^[31]^ Cardiomyocytes express multiple ANT isoforms, with ANT1 being the predominant isoform. Mutations in ANT1, as well as its reduced expression or activity, are linked to severe cardiac diseases.^[32–34]^ The role of ANT2 in cardiac diseases remains unexplored. Our findings revealed increased mRNA expression of SLC25A5 in endomyocardial samples from DCM patients. The potential involvement of SLC25A5 in necroptosis via a mitochondria-dependent pathway aligns with the mitochondrial dysfunction observed in DCM,^[35]^ emphasizing the need for a more in-depth exploration of SLC25A5’s functional mechanisms in this context. Single-cell expression profiling has revealed a predominant presence of SLC25A5 in endothelial cells compared to cardiomyocytes, implying a contributory role of SLC25A5 in both cell types towards DCM pathogenesis. Nevertheless, validation of the expression patterns of SLC25A5 in these cells is essential through empirical validation. Given the diverse nature of DCM, the exclusive representation of congenital DCM cell lines in the GSE184899 dataset warrants further investigation into the extent and significance of SLC25A5’s involvement across various models of DCM pathogenesis.

CNAP1, also known as Calpain-1, is a calcium-regulated non-lysosomal thiol-protease that mediates selective proteolysis of substrates involved in cytoskeletal remodeling and signal transduction. It belongs to the family of calcium-activated cysteine proteases, termed Calpains (CAPNs). CAPN1 is expressed in cardiomyocytes, endothelial cells, smooth muscle cells, and circulating blood cells. In endothelial cells, CAPN1, along with CAPN2 (another CAPN isoform), plays a role in maintaining vascular integrity. However, the enhanced and/or sustained activation of CAPNs have been associated with different pathological conditions including hypertension, atherothrombosis and diabetes.^[36]^ Recent research has highlighted the association between calpain cleavage and the development of heart failure, particularly highlighting the role of increased CAPN1 in myocardial mitochondria in promoting myocardial injury through modulation of mitochondrial superoxide production.^[37]^ CAPN1 is implicated in the cleavage of Junctophilin-2, a crucial protein for maintaining cardiac-coupled compartments, and cardiac substrates such as cTnT, cTnI, and β2-actin, ultimately contributing to heart failure pathogenesis.^[38,39]^ Consequently, targeting CAPN1 emerges as a promising therapeutic approach for heart failure management. Notably, calpain inhibition has shown to enhance outcomes in a heart failure mouse model.^[40]^ CAPN1 has also been linked to phenotypic transformation and calcification of vascular smooth muscle cells, resulting in structural and functional abnormalities in blood vessels. These effects directly or indirectly contribute to cardiac dilation, fibrosis, and functional deterioration.^[31–44]^ The precise mechanisms through which CAPN1 contributes to the initiation and advancement of DCM remain incompletely elucidated. Nevertheless, our research has yielded valuable insights, revealing an upregulated activity of the CAPN1 gene in the context of DCM, particularly within smooth muscle cells. This localized and intensified gene expression pattern strongly implies a crucial role for CAPN1 in the pathogenesis of DCM. Deciphering the increased activity of this gene in smooth muscle cells may offer opportunities for the development of targeted therapeutic strategies for DCM.

INFGR2 plays pivotal a role in IFN-γ signaling within the IFN-γ receptor complex. IFN-γ is a cytokine known for its immunomodulatory properties, stimulating macrophages and T cells and impacting the immune response. Elevated IFN-γ levels can trigger autoimmune-mediated inflammation in cardiac tissues, leading to conditions such as cardiac dysfunction, heart failure, cardiac arrhythmias, or pericarditis.^[45–47]^ While direct evidence linking the IFNGR2 gene to DCM is currently lacking in the literature, its potential involvement in disease progression may stem from the genetic diversity of DCM and the immunoinflammatory mechanism, leading to abnormal activation of the IFN-γ signaling pathway. Further validation through gene sequencing and functional experiments is recommended to confirm its significance, alongside exploring therapeutic strategies targeting the immune pathway.

CAMK2A, also known as CaMKIIα, represents the principal isoform of calcium/calmodulin-dependent protein kinase IIα, predominantly found in the brain with some presence in the heart.^[48,49]^ While CaMKII has been implicated in various aspects of heart failure and arrhythmias, the specific roles of CaMKIIα isoforms in cardiac physiology remain unclear, highlighting the necessity for further research to delineate their impacts on cardiac development, function, and pathology. Interestingly, excessive activation of CaMKII has been shown to disrupt cardiomyocyte calcium dynamics, impacting both systolic and diastolic cardiac function.^[50]^ In contrast, our study reveals a down-regulation of CAMK2A expression in DCM, with predominant expression in fibroblasts, emphasizing the requirement for additional experimental investigations.

BIRC3, also known as cIAP2, is an intracellular anti-apoptotic protein belonging to the IAP family. Its role in cancer is multifaceted, displaying dual functions as both a tumor suppressor and a tumor promoter.^[51]^ Despite its implications in various cancers, the involvement of BIRC3 in cardiovascular disease remains relatively unexplored. In our findings, BIRC3 emerged as a central gene in the bulk-sequencing dataset, demonstrating potential as a valuable diagnostic marker. However, its widespread expression observed in single-cell analysis may have precluded its utility as a diagnostic marker while underscoring its significance as a therapeutic target.

Finally, we utilized the DGIdb to predict drugs for the 5 hub genes, identifying a potential candidate: Calpeptin. As an inhibitor of CAPN1, Calpeptin is envisaged as a therapeutic agent to intervene in the dysregulated expression of CAPN1 in DCM.

Our study is subject to several limitations. Primarily, the reliance on data from public databases introduces potential biases and constraints. The identification of hub genes is based on a small sample size from the GSE128095 dataset, emphasizing the necessity for larger cohort studies to enhance the robustness of the findings. Furthermore, the GSE184899 dataset consists solely of cell lines from individuals with congenital dilated cardiomyopathy, excluding representation from other DCM forms, limiting the understanding of DCM heterogeneity. Therefore, a broader cohort is imperative to fortify the study’s conclusions. Secondly, while bioinformatics analyses offer initial insights, experimental validation is crucial to confirm their accuracy. Therefore, future endeavors should focus on elucidating the biological and molecular functions of these molecules through additional experimental validation. Thirdly, the DGIdb database utilized for drug prediction may exhibit incomplete drug coverage and limited clinical trial correlations. Additionally, additional assessment of the therapeutic efficacy of the candidate compound Calpeptin in DCM animal models is warranted.

## 5. Conclusions

The primary objective of the study was to develop a necroptosis-based diagnostic tool for DCM. Secondary aims included:

1. Identifying key necroptosis-related genes driving DCM pathogenesis through multi-omics bioinformatics analysis;
2. Validating the diagnostic efficacy of these molecular signatures in patient cohorts;
3. Exploring potential therapeutic agents targeting necroptosis pathways.

In summary, 5 crucial necroptosis-related genes (*SLC25A5*, *CNAP1*, *IFNGR2*, *CAMK2A* and *BIRC3*) associated with DCM were identified using comprehensive bioinformatics analysis. A diagnostic model based on these genes was constructed, demonstrating strong predictive performance for early DCM diagnosis and risk assessment. The potential therapeutic drug Calpeptin discovered in this research provides a novel idea for the precise treatment of DCM. Hence, these findings have significant implications for enhancing the diagnosis and identifying novel therapeutic targets for clinical conditions involving DCM. Future research should focus on larger-scale studies and additional experimental validations to elucidate the precise roles of these genes in DCM pathogenesis and to develop tailored treatment strategies based on these molecular targets.

## Acknowledgments

This study used data or information from the GEO database. We thanked the GEO database for the provided data.

## Author contributions

**Conceptualization:** Meng Xia, Hongtao Zhu.

**Data curation:** Meng Xia, Yanan Ji.

**Formal analysis:** Meng Xia, Yanan Ji.

**Investigation:** Meng Xia.

**Methodology:** Meng Xia, Hongtao Zhu.

**Supervision:** Hongtao Zhu.

**Writing – original draft:** Meng Xia, Hongtao Zhu.

**Writing – review & editing:** Meng Xia.
